# Cyanophage Distribution Across European Lakes of the Temperate-Humid Continental Climate Zone Assessed Using PCR-Based Genetic Markers

**DOI:** 10.1007/s00248-021-01783-y

**Published:** 2021-06-05

**Authors:** Aleksandra Jaskulska, Sigitas Šulčius, Mikołaj Kokociński, Judita Koreivienė, Arnoldo Font Nájera, Joanna Mankiewicz-Boczek

**Affiliations:** 1 UNESCO Chair on Ecohydrology and Applied Ecology, Faculty of Biology and Environmental Protection, University of Łódź, 12/16 Banacha, 90-237 Łódź, Poland; 2 Laboratory of Algology and Microbial Ecology, Nature Research Centre, 2 Akademijos, 08412 Vilnius, Lithuania; 3 Department of Hydrobiology, Adam Mickiewicz University, 6 Uniwersytetu Poznańskiego, 61-614 Poznań, Poland; 4 European Regional Centre for Ecohydrology of the Polish Academy of Sciences, 3 Tylna, 90-364 Łódź, Poland

**Keywords:** Cyanobacteria, Cyanophage, *Psb*A, *Nbl*A, *g91*

## Abstract

Studies of the diversity and distribution of freshwater cyanophages are generally limited to the small geographical areas, in many cases including only one or few lakes. Data from dozens of various lakes distributed at a larger distance are necessary to understand their spatial distribution and sensitivity to biotic and abiotic factors. Thus, the objective of this study was to analyze the diversity and distribution of cyanophages within the infected cells using marker genes (*psb*A, *nbl*A, and *g91*) in 21 Polish and Lithuanian lakes. Physicochemical factors that might be related to them were also analyzed. The results demonstrated that genetic markers representing cyanophages were observed in most lakes studied. The frequently detected gene was *psb*A with 88% of cyanophage-positive samples, while *nbl*A and *g91* were found in approximately 50% of lakes. The DNA sequence analyses for each gene demonstrated low variability between them, although the *psb*A sequences branched within the larger cluster of marine *Synechoccocuss* counterparts. The principal component analysis allowed to identify significant variation between the lakes that presented high and low cyanobacterial biomass. The lakes with high cyanobacterial biomass were further separated by country and the different diversity of cyanobacteria species, particularly *Planktothrix agardhii*, was dominant in the Polish lakes and *Planktolyngbya limnetica* in the Lithuanian lakes. The total phosphorous and the presence of cyanophage genes *psb*A and *nbl*A were the most important factors that allowed differentiation for the Polish lakes, while the pH and the genes *g91* and *nbl*A for the Lithuanian lakes.

**Supplementary Information:**

The online version contains supplementary material available at 10.1007/s00248-021-01783-y.

## Introduction

Cyanophages, viruses infecting cyanobacteria, are numerous biologically active entities in aquatic ecosystems and play an important role in determining host population diversity, dynamics, and evolution [1–4]. Most of the currently known cyanophages are members of *Myoviridae* (myocyanophages), *Siphoviridae* (siphocyanophages), and *Podoviridae* (podocyanophages) families [5–7]. Among these, the diversity of myocyanophages is probably the most well represented in public databases to date. However, some studies indicate that sipho- and podoviruses might exhibit higher actual diversity compared to members of *Myoviridae* [8–10]. Cyanophage distribution is often correlated with the distribution of their hosts, and their abundance changes in time and space [1, 11, 12]. Cyanoprokaryota (cyanobacteria), including scum-forming genera *Microcystis*, single-celled members of *Cyanobium* or *Synechococcus*, and filamentous species belonging to *Lyngbya*, *Oscillatoria*, *Planktothrix*, and *Phormidium*, are widely distributed photosynthetic organisms [13]. Among them, *Microcystis* and *Synechococcus* are two of the most described in the context of susceptibility to viral infections [14–16]. Moreover, the environmental studies indicated that the distribution and diversity of cyanophages might be directly or indirectly (through the host) affected by physicochemical agents [17–20]. According to Finke and Suttle [21], the diversity of the marine phage community depends on a promoted variety of environmental factors including salinity, temperature, and concentration of nutrients, followed by water column mixing. Solar radiation may damage viral particles and negatively influence infection efficiency as described by Wilhelm et al. [22] in the marine environment. The studies of freshwater cyanophages conducted by Cheng et al. [23] also showed that decay of their infectivity was correlated with UV intensity. The cyanophage composition was also found to be influenced by seasonal variations and water column depth as described by Hurwitz et al. [24] based on ocean metagenomics studies. Despite the growing number of researches on cyanophages, the information about their complex diversity and distribution in freshwater remains insufficient.

Recently, Finke and Suttle [21] showed that a specific individual gene (*gp43*), that is, used as a genetic marker to assess virus diversity, can highly reflect the variation observed by the whole genome and gene content comparisons. The diversity of cyanophages can be assessed using phage group/clade-specific molecular markers such as those encoding major capsid protein, portal protein, tail sheath protein, and DNA polymerase [25–27]. The host-derived cyanophage auxiliary metabolic genes (AMGs) are also widely used to assess cyanophage diversity and distribution. For example, genes *psb*A and *nbl*A, which encode the D1 protein of photosystem II (PSII) and nonbleaching protein A, respectively [16, 28]. The *psbA* genes were reported as highly prevalent among some marine myo- and podocyanophages (clade A) which infected *Prochlorococcus* and *Synechococcus* [4, 29–31]. The *psb*A genes were also identified in some freshwater cyanophages (e.g., *Synechococcus* phage S-CRM01); however, their prevalence in this aquatic environment is less known [4, 32]. The *nbl*A gene was also proposed as the genetic marker and was found in freshwater cyanophages infecting *Microcystis* and then *Planktothrix* [15, 16, 33]. However, some studies indicated that this gene is highly conserved and thus tends to underrepresent genetic diversity [15, 16, 33–35]. The structural gene *g91* encoding tail sheath protein in cyanophages infecting *Microcystis aeruginosa* was used to assess their diversity. Based on the comparative analysis of this gene, three major genotypes were distinguished and their spatial and temporal distribution have been tracked [36, 37].

**Fig. 1:**
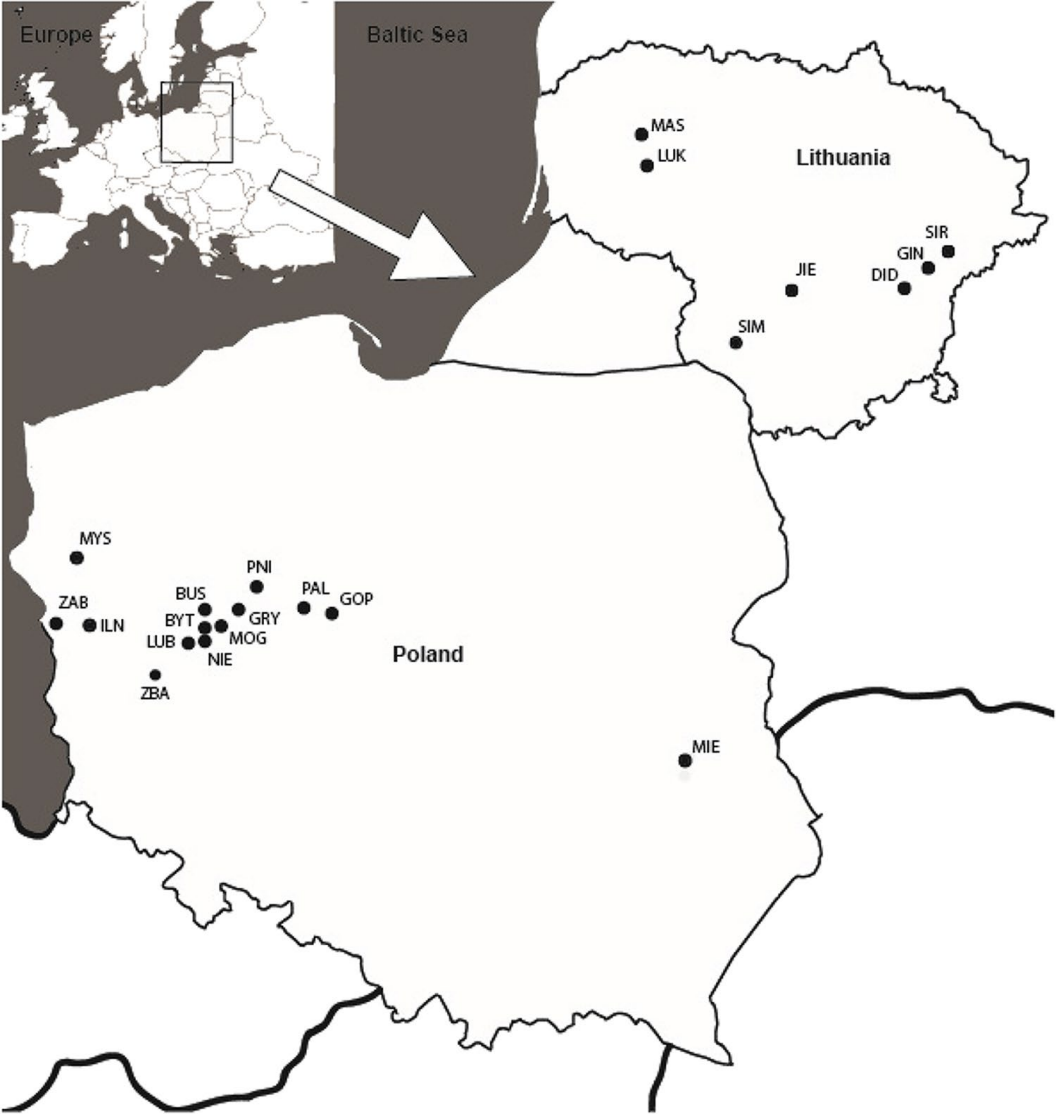
Study site of lakes in Poland and Lithuania

The occurrence and monitoring of freshwater cyanophages based on the abovementioned genes were conducted in situ in several different ecosystems in Japan [28, 36–39], China [16, 40], France [41], the USA [4], Poland [42], and Canada [43]. However, most of the studies conducted so far were limited to one or two water bodies and none of them referred to the occurrence and diversity of freshwater cyanophages, including a larger geographical area. Presuming that differences in cyanophage community compositions would increase with geographic distance (distance-decay hypothesis) and that spatial distribution patterns is a result of interplay between cyanophages, cyanobacteria, and environmental conditions; one could expect that observed cyanophage diversity would reflect the area surveyed. Therefore, the present study aimed to determine the diversity and spatial distribution of active cyanophages community, which were infecting cyanobacteria, from an extensive area spanned over two countries. Towards this aim, we analyzed sequence diversity of three different marker genes (*psb*A, *nbl*A, and *g91*) in 21 lakes of the temperate-humid continental climate zone (Poland and Lithuania), in an area with a span of approx. over 200,000 km^2^ (Fig. 1). Besides, we assessed the relationship between the occurrence of marker genes, their sequence diversity, cyanobacterial communities composition, and environmental variables. Such information could be helpful to explore the potential linkage between cyanophages and their host—cyanobacteria, their spatial distribution between waterbodies, and sensitivity on environmental factors.

## Materials and Methods

### Source of Material

Samples were collected from 14 Polish and 7 Lithuanian lakes situated in the temperate-humid continental climate zone. They were the subject of research on cyanobacteria in our previous publication [44]. Samples were collected from the following: Lubosińskie (LUB), Bytyńskie (BYT), Buszewskie (BUS), Pniewskie (PNI), Myśliborskie (MYS), Pakoskie (PAL), Grylewskie (GRY), Mogileńskie (MOG), Niepruszewskie (NIE), Ilno (ILN), Gopło (GOP), Żabiniec (ZAB), Zbąszyńskie (ZBA), Miejskie (MIE), Jieznas (JIE), Širvys (SIR), Gineitiškės (GIN), Didžiulis (DID), Mastis (MAS), Lūkstas (LUK), and Simnas (SIM) (Fig. 1, Fig. S1). They represent fertile lakes from meso-eutrophic to hypertrophic with high phytoplankton diversity (Table S1 ).

### Sampling

Samples were collected from the central part of the lake in August 2013 and July–August 2014 (Fig. 1, Fig S1). Integrated phytoplankton samples were collected from the epilimnion in stratified lakes or from the surface water layer in polymictic lakes from one sampling station using a water sampler. Approximately, 300 mL of water samples were collected to aseptic plastic bottles as integrated water probes from the water column (e.g., mixed water from samples taken every 1 m deep) during the early afternoon (Fig. 1, Fig. S1). The 1-L phytoplankton samples were preserved with acidified Lugol’s solution with a final concentration of 1% immediately after sampling. The samples were transferred to the laboratories and stored under cool and dark conditions until they were analyzed.

### Measurements and Analyses of Physicochemical Parameters

Water temperature, pH, and conductivity were determined in situ using a multiparameter probe. Integrated water samples were collected for chemical analyses. The water samples were analyzed for total nitrogen (TN) and total phosphorus (TP) with a HACH spectrophotometer [45, 46].

### Analysis of Cyanobacterial Composition

Phytoplankton samples were sedimented in 1-L glass cylinder for 48 h, gently decanted off, and the final sample volume of 20–30 mL was used for further analysis. Cyanobacterial species identification [47–49] and counts were conducted using light microscopes under × 400 magnification. The enumeration of specimens was carried out in 100–150 fields of Fuchs-Rosenthal chamber, which ensured that at least 400 specimens were counted to reduce the error to less than 10%. A single cell, a coenobium, or a filament represented one specimen in the analysis. The biovolume of each species was determined through a volumetric analysis of cells using geometric approximation and expressed as a wet weight following Wetzel and Likens [50].

### Isolation and Amplification of Genes

Freshwater samples in the volume of 100 mL each were filtered onto 0.45 µm nitrocellulose membrane filters (Millipore, USA). Subsequently, filters containing cell fraction were inserted in the 2 mL of lysis buffer (400 mM NaCl, 40 mM EDTA, 0.75 M sucrose, and 50 mM TRIS–HCl; pH 8.3), then stored at − 20 °C before DNA extraction.

DNA was isolated from stored filters according to hot phenol-mediated extraction described by Giovannoni et al. [51] with minor changes described by Mankiewicz-Boczek et al. [52] including the modification of centrifugation speed (13,000* g*) and the final concentration of proteinase K (275 µg mL^1−^). The extracted nucleic acid was used as a template for molecular analyses of the genes for general presence of cyanobacteria, 16S rRNA (258 bp), and the specific presence of *Microcystis* genus, 16S rRNA (250 bp), and for cyanophages—*psb*A (740 bp), *nbl*A (200 bp), and the *g91* (*g91*_S – 132 bp, and *g91*_L – 206 bp) which together cover all three genotype groups distinguish by Kimura-Sakai et al. [37]. All nucleotide primers and parameters of PCR are described in Table S2 and Table S3 (supplementary materials).

### Sequencing of Cyanophage Genes

DNA samples for nucleotide Sanger sequence analyses (Table 1) of genes: *psb*A, *nbl*A, and *g91* were chosen based on the good quality PCR amplicons. In consequence, the *psb*A, *nbl*A, *g91*_S, and *g91*_L were analyzed and deposited in Genbank database for six (LUB, BUS, PAL, PNI, GIN, and ILN (accession numbers: MW853986 to MW853991, respectively)), six (BUS, SIM, BYT, PAL, PNI, and GIN (accession numbers: MW853992 to MW853997, respectively)), nine (LUB, MIE, BUS, SIM, BYT, PAL, PNI, JIE, and GIN (accession numbers: MW853982 to MW853985, respectively)), and four (SIM, BYT, JIE, and GIN (accession numbers: MW853992 to MW853997, respectively)) lake samples, respectively. To prepare samples for sequencing, the selected DNA samples were amplified with the use of Pfu DNA polymerase (forming blunt-end; Thermo Scientific) according to producer procedure and PCR conditions showed in Table S3. The specific primer sequences, chemical concentration, and amplification program for PCR can be found in Table S2 and Table S3. Obtained PCR products were purified with the use of QIAGEX® II Gel extraction Kit (QIAGEN), cloned into pJET1.2/blunt vector (Thermo Scientific), and sequenced (Genomed S.A.). The obtained forward sequences were improved by reverse complementation and the primer sequences were clipped out with the use of a BioEdit Sequence Alignment Editor (version 7.2.5).

**Table 1:** Presence of cyanobacteria and cyanophage amplicons, and PCA group results of studied lakes

Country	Lakes	Cyanobacteria 16S rRNA*	Cyanophages *psb*A	*Microcystis* spp. 16S rRNA	*nbl*A**	*g91_*S**	*g91*_L**	PCA groups
Poland	LUB	+	+	+	+	+	+	A
	BYT	+	+	+	+	+	+	A
	BUS	+	+	+	+	+	na	A
	PNI	+	+	+	+	+	+	A
	PAL	+	+	+	+	+	na	A
	MIE	+	na	+	+	+	na	-
	NIE	+	+	+	na	na	na	-
	MYS	+	na	+	na	na	+	C
	GRY	+	+	na	na	na	na	C
	MOG	+	na	+	na	na	na	C
	ILN	+	+	na	na	na	na	C
	GOP	+	+	na	na	na	na	C
	ZAB	+	na	+	na	na	na	C
	ZBA	+	+	+	na	na	na	C
Lithuania	DID	+	+	+	na	na	na	C
	SIR	+	na	+	na	na	na	C
	JIE	+	+	+	na	+	+	B
	SIM	+	+	+	+	+	+	B
	GIN	+	+	+	+	+	+	B
	MAS	+	na	+	na	na	na	-
	LUK	+	na	+	na	na	na	-

+ , presence of amplicon; na no amplicon;*, universal 16S rRNA gene sequence for cyanobacteria; **, gene fragments specific for Microcystis cyanophages; -, not grouped

A phylogenetic tree was constructed for the *psb*A gene. A cluster was performed, separately, for cyanophage and cyanobacterial sequences with 90% similarity. The cyanophages and cyanobacteria sequences were taken from the NCBI non-redundant database (https://blast.ncbi.nlm.nih.gov/Blast.cgi). Then, the sequences were aligned with the use of MAFFT-DASH and the tree was constructed with the use of RAxML NG.

In case of the search for similar sequences of gene fragments (*nbl*A and *g91*) shorter than 200 bp, the online Local Alignment Search Tool (BLAST), based on data from the following databases: the NCBI non-redundant sequence database (https://blast.ncbi.nlm.nih.gov/Blast.cgi), JGI virus public database (https://img.jgi.doe.gov/), and viruSITE integrated database (www.virusite.org), were used.

### Statistical Analysis

The principal component analysis (PCA) was used to evaluate the spatial distance between 21 lakes according to the total abundance of different cyanobacterial species, the occurrence of cyanophage genes (*psb*A, *nbl*A, *g91*_S and, *g91*_L), and the environmental factors including nutrients (TP and TN) and physicochemical parameters (temperature, pH, and conductivity). All data were transformed to avoid skewed distributions with the subtraction of the mean and the division with the standard deviation ((x-mean)/Sd). Groups were defined according to the number of genes detected for each lake. The one-way ANOVA and Tukey’s tests were used to measure significant differences between the groups with the scores obtained for the PC1 and PC2 (Table S4). The PCA was performed with PAST 4.03 [53]. Levine’s test was used to check homogeneity of variance from the means. The proposed statistical analysis is used to condensate multivariate databases often obtain in environmental studies, allowing to identify the most important factors that explain the highest variance within a set of samples [54].

## Results and Discussion

Cyanophages are specialized to infect cyanobacteria and could play an important role in modulating harmful blooms. As cyanophage distribution was found related to the occurrence of their hosts [1, 11, 12], it is needed to obtain knowledge of the cyanobacteria composition and factors influencing their growth in the study area. Analysis of cyanobacteria—potential virus host—indicated that their 16S rRNA gene was found in all studied lakes (Table 1). The total cyanobacteria biomass varied from 0.04 to 40.47 mg L^−1^ (Table S1). Filamentous cyanobacteria from the genera *Aphanizomenon*, *Cuspidothrix*, *Dolichospermum*, *Limnothrix*, *Planktolyngbya*, *Pseudanabaena*, *Planktothrix*, or *Raphidopsis* were among the dominants in most studied lakes. Additionally, *Microcystis* was among the dominant genera (0.57–1.35 mg L^−1^) in three lakes based on the microscopic analysis, and their overall presence was confirmed in 18 lakes according to the genetic analysis—16S rRNA (Tables 1 and 2, Table S1).

**Table 2:** Comparison of the variables among tree lake groups distinguished in PCA

Group	Lakes	pH	Cond (uS*cm^−1^)	Temp (°C)	TN (mg*l^−1^)	TP (mg*l^−1^)	TN:TP ratio	CYAN (mg l^−1^)	Dominant cyanobacteria and their biomass (mg l^−1^)*
A	LUB, BYT, BUS, PNI, PAL	8.76 ± 0.17	627 ± 73	22.9 ± 0.78	1.97 ± 0.29	0.270 ± 0.13	8.8 ± 4.4	14.21 ± 15.18	*Planktothrix agardhii* 1.85–38.33 (av. 15.67), *Aphanizomenon gracile* 2.28–5.32 (av. 3.80), *Limnothrix* spp*.* 0.91–2.20 (av. 1.75), R*aphidiopsis raciborskii* 1.43, *Jaaginema subtilissimum* 0.69, *Microcystis aeruginosa* 1.35
B	JIE, GIN, SIM	8.72 ± 0.26	320 ± 66	23.7 ± 6.5	1.51 ± 0.23	0.063 ± 0.015	25.3 ± 9.0	16.55 ± 9.86	*Aphanizomenon gracile* 0.63–5.99 (av. 3.27), *Planktolyngbya limnetica* 2.96–3.41 (av. 3.19), *Pseudanabaena limnetica* 3.80*, Microcystis viridis* 0.89, *Woronichinia naegeliana* 2.47, *Cuspidothrix issachenkoi* 15.88
C	ZBA, GOP, GRY, ILN, MYS, ZAB, DID, SIR, MOG	8.22 ± 0.55	521 ± 109	21.1 ± 3.3	1.88 ± 0.77	0.087 ± 0.073	42.9 ± 45.5	8.60 ± 12.08	*Planktothrix agardhii* 8.58–36.35 (av. 14.22), *Aphanizomenon gracile* 0.47–0.81 (av. 0.64), *Pseudanabaena limnetica* 0.73–0.89 (av. 0.81), *Jaaginema subtilissimum* 0.01–0.14 (av. 0.08), *Aphanizomenon flos-aquae* 0.09, *Raphidiopsis raciborskii* 0.06, *Synechococcus salinarum* 0.15, *Dolichospermum lemmermannii* 0.42, *Dolichospermum planctonicum*0.44, *Microcystis* sp. 0.57*, Limnothrix redekei* 0.04, *Aphanizomenon kelbanii* 0.73, *Planktolyngbya* sp. 2.27, *Pseudanabaena limnetica* 1.46

A, presence of three genes; B, presence of two–three genes; C, presence of one or none gene

The study area (Fig. 1) was represented by the temperate-humid continental climate zone characterized by hot summers [55] together with water parameters which are shown in the following ranges: water pH 7.4–9.01, water temperature 16.3–27.8 °C, and conductivity 251–729.1 µS cm^−1^. While total nitrogen and total phosphorus concentrations varied between 0.85–7.5 and 0.02–0.47 mg L^−1^, respectively (Table S1), such parameters, conducive to eutrophication, ensured background and favored the development of cyanobacteria [56, 57].

According to the authors’ knowledge, the presented studies are the first which refer to the relationship between the occurrence of all three cyanophage marker genes simultaneously (*psb*A, *nbl*A, and *g91*), their sequence diversity, cyanobacterial communities composition, and environmental variables from the freshwater environment of an extensive area (approx. over 200,000 km^2^). Moreover, the results described below confirmed that the environmental factors, most likely local, may have an important role in shaping the genetic variation in phages.

### Cyanophages Occurrence and Diversity

The cyanophage genes (*psb*A, *nbl*A, or *g91*) presented in host cells were detected in 16 from the 21 studied lakes (Fig. 1, Table 1). The lack of amplification of selected marker genes for cyanophages in some lakes, despite the presence of their potential hosts, may have been related to the number of the genes below the detection limit or used genetic markers not targeting the different phage communities, present in the lakes studied. According to Schrader et al. [58], the PCR inhibitors should be also taken into consideration.

**Fig. 2:**
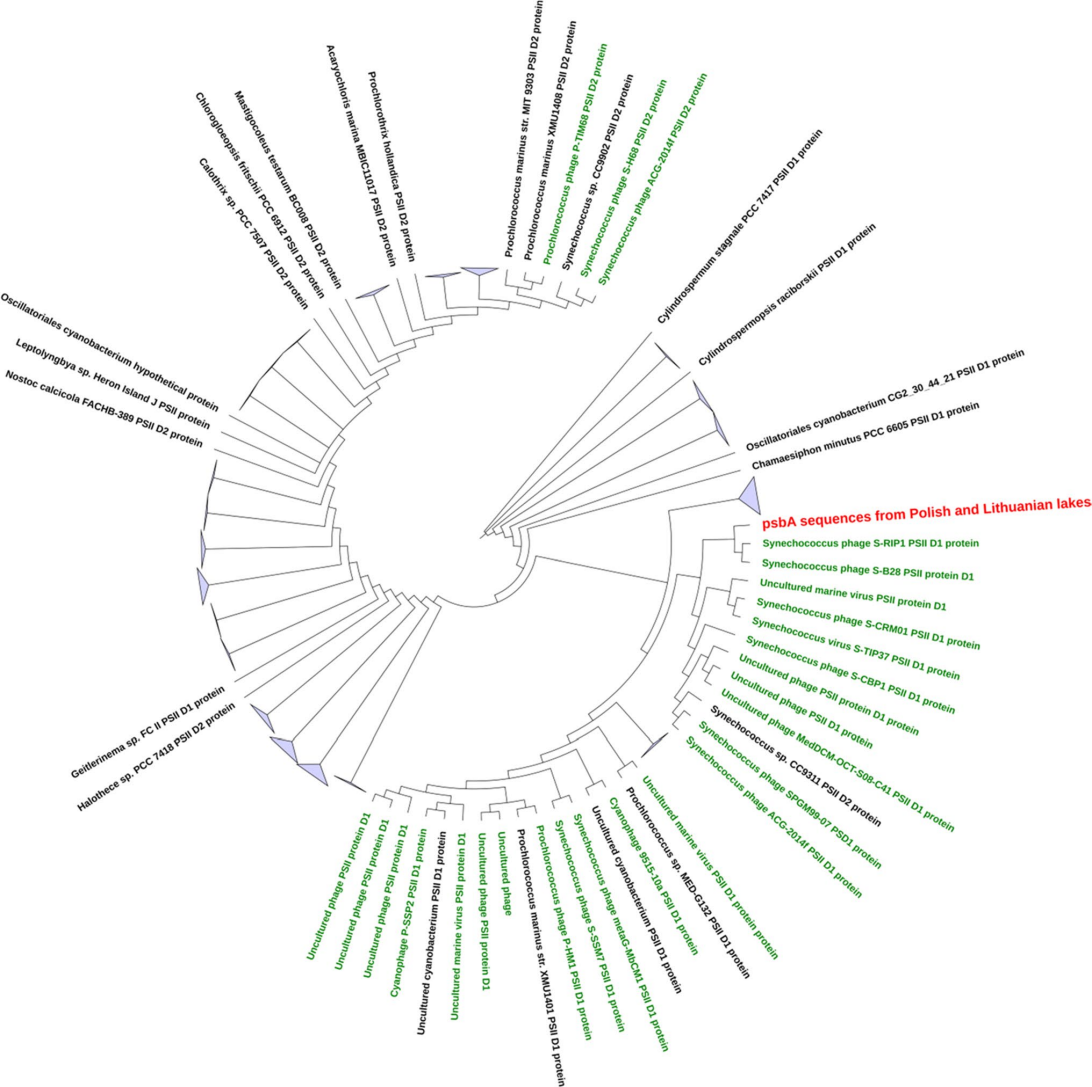
Phylogenetic tree of *psb*A sequence fragment alignment among different cyanophages and cyanobacteria

**Fig. 3:**
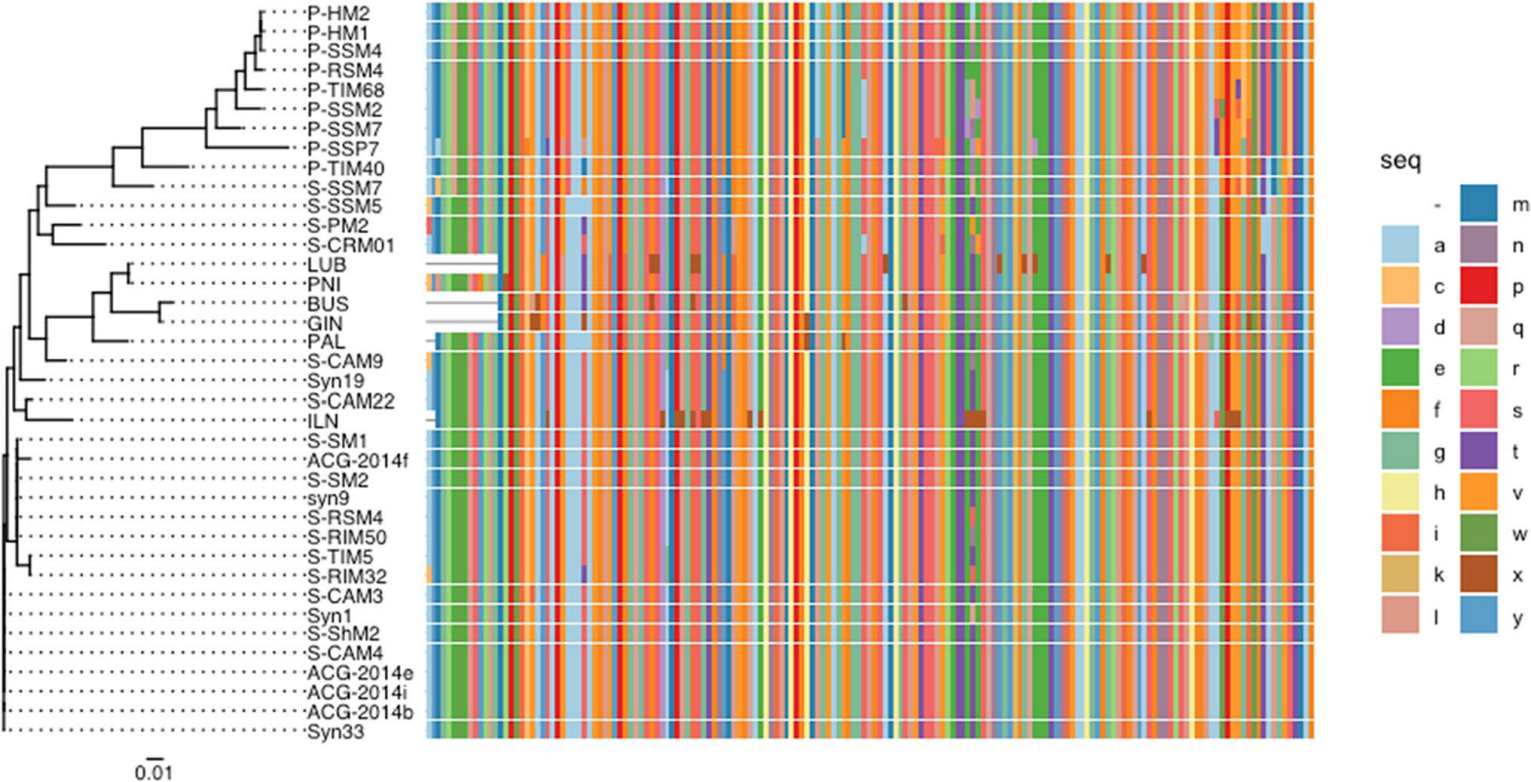
Phylogenetic tree and multiple amino acid sequence alignment of psbA gene fragments among different cyanophages

The *psb*A was found in 88% cyanophage-positive samples (Table 1). Its DNA sequences were found between 75 and 98% of similarity for five Polish lakes (LUB, PNI, BUS, PAL, and ILN) and one Lithuanian lake (GIN). The variants with the highest similarity level were observed between LUB-PNI (98%) and BUS-GIN (95%). The *psb*A sequence of ILN had the lowest level of similarity (75–78%) with the analyzed sequences. Although all *psbA* sequences observed in this study branched within the larger cluster of marine cyanophages, they also grouped more closely to each other than to their marine counterparts (Fig. 2). Most of the *psbA* sequences showed 95–100% similarity to each other (data were not shown), with the only exception of Lake ILN (Fig. 3). The *psb*A sequences were intermixed, indicating that there were no differences in the distribution of cyanophages between distant lakes. Therefore, the higher divergence of ILN from other lakes may suggest that other, most likely local, factors might be responsible for the diversity of the cyanophage community, whereas the *psb*A sequences from Polish lake ILN appeared to be the most similar with marine *Synechococcus* myocyanophage genome (S-CAM22) (Fig. 3). As it was described by Dreher et al. [26], the *psbA* similarity to the marine counterparts was also confirmed within *Synechococcus*-specific S-CRM01 cyanophage, isolated from freshwater Copco Reservoir (Northern California, USA). The high similarity of freshwater cyanophages *psb*A to marine cyanomyoviruses was also found in East lake (China) by Ge et al. [59]. Moreover, the *psb*A of novel freshwater Ma-LEP *Microcystis* podocyanophage, isolated from Erie lake (USA) by Jiang et al. [4], also presented high sequence similarity with marine S-CBP4 *Synechococcus* podocyanophage. The above results, of freshwater *psb*A sequence similarities to their marine counterparts, indicated that this genetic marker can be used to study the diversity among freshwater and marine phages as already described by Chenard and Suttle [32].

The *nbl*A, *g91*_S, and *g91*_L *Microcystis* cyanophage genes were found in 50%, 56%, and 44% of cyanophage-positive samples, respectively (Table 1). All *nlb*A sequences observed in this study from the analyzed samples (BYT, BUS, PNI, PAL, GIN, and SIM) if compared to each other showed high similarity, ranging from 88 to 99%. The highest level of sequence similarity (96%) was found between two Polish lakes—BYT and BUS—and between two Lithuanian lakes—GIN and SIM. The *nbl*A sequences from analyzed samples were highly similar (> 90%) with their corresponding gene fragments of uncultured *Myoviridae* phages (AB812972.1 and AB812972) and MaMV-DC (KF356199.1) The literature data indicated that the *nbl*A gene is highly conserved and, hence, may underrepresent the existing diversity among cyanophages [34]. The *g91_*S sequences obtained from six Polish lakes (LUB, BYT, BUS, PNI, PAL, and MIE) and three Lithuanian lakes (JIE and SIM) were similar in the range of 90–97% between them. Only GIN showed the lowest similarity (80–85%) when compared to all other sequences. Whereas the *g91*_L from the one Polish (BYT) and three Lithuanian lakes (JIE, GIN, and SIM) were similar in the range of 95–96%, the *g91*_S and *g91*_L sequences from this study were convergent (> 91%) with their counterparts in culturable (MaMV-DC, KF356199.1; Ma-LMM01, AB231700.1) and unculturable (MH117957.1) *Microcystis* cyanophages. The above results might indicate the presence of Ma-LMM01-like phages within investigated lakes, as it was also showed in the Bay of Quinte (a Lake Ontario, Canada) by Rozon and Short [43] or Sulejowski Reservoir (Poland) by Mankiewicz et al. [42]. In the case of lakes where there was no positive detection of *nbl*A and *g91* genes represented Ma-LMM01-like phages, it is also possible that other *Microcystis*-specific phages occur, which genomes were not characterized yet.

### Environmental Variables

**Fig. 4:**
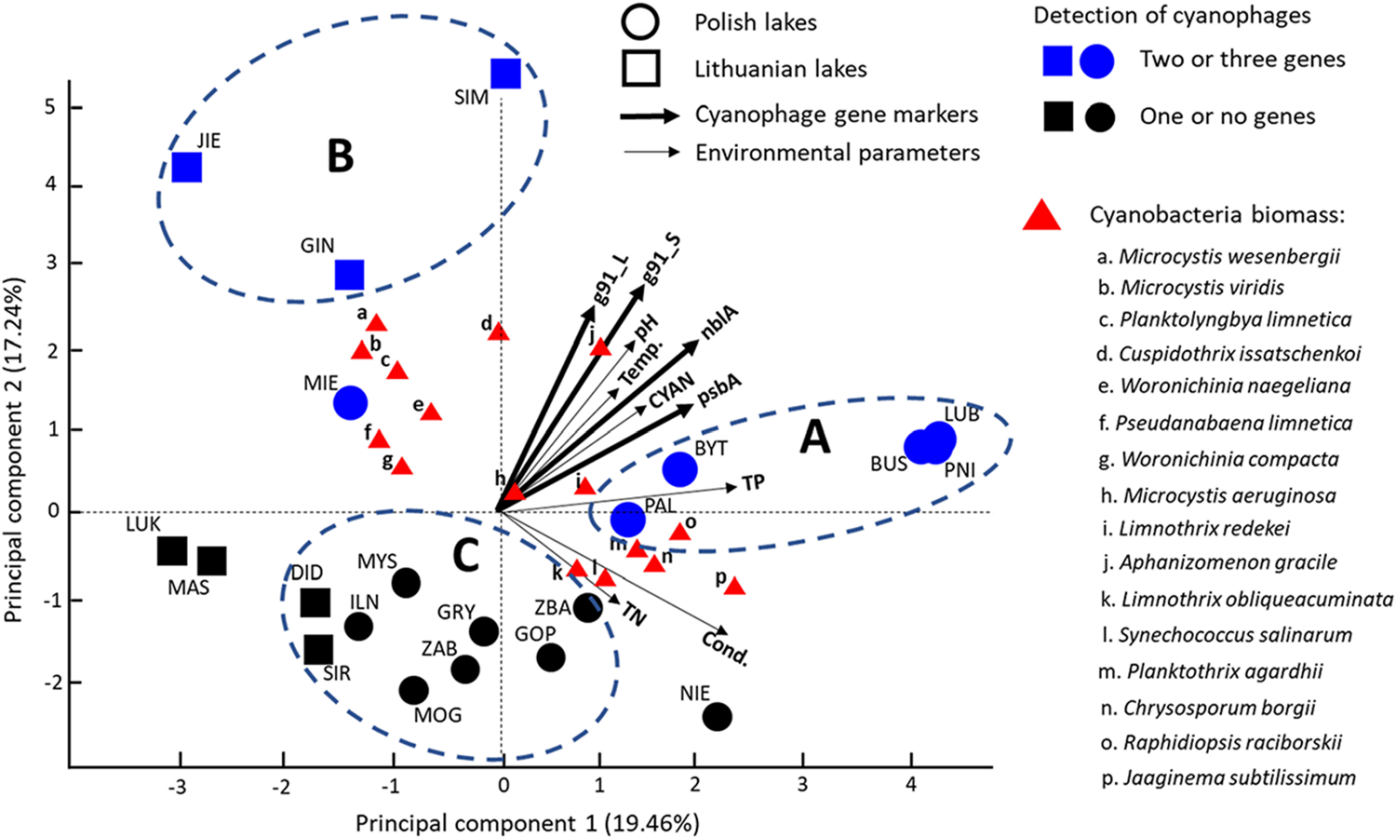
Principal component analysis (PCA) based on environmental–physicochemical variables, diversity, and biomass of cyanobacterial species and cyanophage genes’ presence for Polish and Lithuanian lakes

Principal component analysis (PCA) showed the relationships between cyanophages, cyanobacteria, and the physicochemical parameters of water (Fig. 4). The PC1 and PC2 represented up to 36.7% of the total variance of the observations (19.46% and 17.24%, respectively; see also Fig. S3). The PCA scores and loadings (estimated with the Pearson correlation [*r*]) are described in the supplementary material Table S4 and S5, respectively. The PCA grouped lakes into three different clusters: groups A including only Polish lakes (LUB, PNI, BUS, BYT, and PAL), B including only Lithuanian lakes (SIM, GIN, and JIE), and group C (DID, SIR, ILN, MYS, MOG, ZAB, GRY, GOP, and ZBA) including both Polish and Lithuanian lakes. In groups A and B, two or three cyanophage genetic markers were detected while group C consisted of lakes with only one or none of the studied genes (Table 2, Fig. 4). Group A was significantly segregated from groups B and C (*p* = 4.76 × 10^−4^ and 2.2 × 10^−4^, respectively; see Table S6). The PC1 presented the highest positive correlations with the TP and conductivity (*r* = 0.71 and 0.70, respectively), followed by the occurrence of cyanophage genes—*nbl*A and *psb*A (*r* = 0.56 and 0.52, respectively) (see Table S5). These results suggested that the abovementioned factors could be important variables contributing to the spatial distancing between the Polish and Lithuanian lakes and favored the development of particular cyanobacteria [56, 57], which can differ in A and B groups analyzed (Fig. 4). The modest relationship between the abundance of some viral genes and TP was indicated for the Bay of Quinte by Rozon and Short [43]. Moreover, TP as one of the most important parameters for the regulation of cyanobacterial occurrence could directly influence in their development and thus becoming available to phages for the genome replication process inside the host cell [60, 61].

Whereas the Lithuanian lakes in group B were significantly differentiated from group C by the vertical component—PC2 (*p* = 6.41 × 10^−9^; Table S7 and Fig. 4), which could be explained by the high positive correlations observed between the PC2 and the cyanophage genes—g91_S, g91_L, and *nbl*A (*r* = 0.79, 0.69, and 0.61, respectively), followed by the pH (*r* = 0.47) (see Table S7), cyanophages have a wide range of pH tolerances; however, a decrease in pH below the host’s optimal requirements may directly affect the host’s cells homeostasis and thus negatively affect the intracellular cyanophage replication process [20]. Thus, group C was characterized not only with the lowest detection of cyanophage genes, but also the lowest values of environmental factors, and therefore, was found negatively scored in the PCA (Fig. 4, Table S4).

The *psb*A sequences presented in LUB, PNI, BUS, PAL, and GIN lakes were aligned close to each other within the phylogenetic tree, with exception of ILN lake (Fig. 3). While after comparing their presence with physicochemical factors as part of the PCA analysis (Fig. 4), the mentioned *psb*A sequences with high similarity were divided into two groups: A (LUB, PNI, BUS, and PAL) and B (GIN). The separateness of *psb*A ILN based on its higher sequence divergence was also reflected within PCA results, as the one which was subjected to group C (Fig. 4). This observation might confirm the important role that the environmental factors, most likely local, may have in shaping the genetic variation in phages.

As it was shown, different cyanobacterial species were subjected to different groups highlighted with the use of PCA analysis (Fig. 4, Table S1). For instance, *Planktothrix agardhii* (average biomass 15.6 mg L^−1^) was a characteristic-dominant species in group A, *Planktolyngbya limnetica* (3.2 mg L^−1^) in group B (Fig. 4), whereas *Aphanizomenon gracile* was the dominant species found in groups A and B with average biomass of 3.27 mg L^−1^ and 3.80 mg L^−1^, respectively, and had five times lower biomass in the group C (0.64 mg L^−1^) (Fig. 4, Table S1). Observed species differentiation might result from the influence of different physicochemical factors. For example, lakes from group A where *P. agardhii* was a dominant species were positively related to TP which is in line with previous studies that demonstrated domination of this cyanobacterium in hypertrophic lakes with high concentrations of phosphorus [62, 63]. Also, *A. gracile* is a common dominant species in temperate lakes adapted to various types of environmental and nutritional conditions [64–66]. However, the cyanobacteria composition represented by total biomass was found to rather enhance the cyanophage genes occurrence (Table 2, Table 1S) than single species highlighted within PCA results. It was observed that in the lakes where two–three cyanophage genes were determined, also cyanobacteria biomass was two–three times higher (Table 2, Table 1S).

## Conclusions

The research of cyanophages based on the amplification of *psb*A, *nbl*A, and *g91* genes confirmed their occurrence in most of the studied lakes. The DNA sequences obtained for each gene showed a high similarity between them. Also, the similarity to their marine *Synechococcus* myocyanophage (*psb*A) and freshwater *Microcystis* myocyanophages (*nbl*A and *g91*) counterparts was confirmed. Furthermore, the *psbA* revealed higher diversity, in comparison to the *nblA* and *g91* genes. In consequence, no clear distribution pattern for cyanophages can be detected. The principal component analysis showed that TP and pH could be important environmental parameters differentiating the sampling sites between the lakes and might directly or indirectly (by cyanobacteria) influence the occurrence of cyanophages.

## Supplementary information

ESM 1 (DOCX 180 kb)

## Data Availability

-DNA sequences: Genbank accessions AB812972.1, AB812972, KF356199.1, KF356199.1, AB231700.1, MH117957.1, MW853973, MW853974, MW853975, MW853976, MW853977, MW853978, MW853979, MW853980, MW853981, MW853982, MW853983, MW853984, MW853985, MW853986, MW853987, MW853988, MW853989, MW853990, MW853991, MW853992, MW853993, MW853994, MW853995, MW853996, MW853997.

## References

[1] Suttle CA, Chan AM (1994). Dynamics and distribution of cyanophages and their effect on marine Synechococcus spp. Appl Environ Microbiol.

[2] Ackermann H-W (2003). Bacteriophage observations and evolution. Res Microbiol.

[3] Hargreaves KR, Anderson NJ, Clokie MRJ (2013). Recovery of viable cyanophages from the sediments of a eutrophic lake at decadal timescales. FEMS Microbial Ecol.

[4] Jiang X, Ha C, Lee S, Kwon J, Cho H, Gorham T, Lee J (2019). Characterization of cyanophages in Lake Erie: interaction mechanisms and structural damage of toxic cyanobacteria. Toxins.

[5] Deng L, Hayes PK (2008). Evidence for cyanophages active against bloom-forming freshwater cyanobacteria. Freshw Biol.

[6] Li S, Ou T, Zhang Q (2013). Two virus-like particles that cause lytic infections in freshwater cyanobacteria. Virologica Sinica.

[7] Hou W, Wang S, Briggs BR, Li G, Xie W, Dong H (2018). High diversity of *Myocyanophage* in various aquatic environments revealed by high-throughput sequencing of major capsid protein gene with a new set of primers. Front Microbiol.

[8] Hendrix RW (2003). Bacteriophage genomics. Curr Opin Microbiol.

[9] Grose JH, Casjens SR (2014). Understanding the enormous diversity of bacteriophages: the tailed phages that infect the bacterial family *Enterobacteriaceae*. Virology.

[10] Olsen NS, Kot W, Junco LMF, Hansen LH (2020). Exploring the remarkable diversity of *Escherichia coli* phages in the Danish Wastewater Environment. Viruses.

[11] Waterbury JB, Valois FW (1993). Resistance to co-occurring phages enables marine *Synechococcus* communities to coexist with cyanophages abundant in seawater. Appl Environ Microbiol.

[12] Sieradzki E, Ignacio-Espinoza JC, Needham D (2019). Dynamic marine viral infections and major contribution to photosynthetic processes shown by spatiotemporal picoplankton metatranscriptomes. Nat Commun.

[13] Paerl HW, Otten TG, Kudela R (2018). Mitigating the expansion of harmful algal blooms across the freshwater-to-marine continuum. Environ Sci Technol.

[14] Dillon A, Parry JD (2008). Characterization of temperate cyanophages active against freshwater phycocyanin-rich *Synechococcus* species. Freshw Biol.

[15] Yoshida T, Nagasaki K, Takashima Y, Shirai Y, Tomaru Y, Takao Y, Sakamoto S, Hiroishi S, Ogata H (2008). Ma-LMM01 infecting toxic *Microcystis* aeruginosa illuminates diverse cyanophage genome strategies. J Bacteriol.

[16] Ou T, Gao X-C, Li A-H, Zhang Q-Y (2015). Genome analysis and gene *nbl*A identification of *Microcystis aeruginosa* myovirus (MaMV-DC) reveal the evidence for horizontal gene transfer events between cyanomyovirus and host. J Gen Virol.

[17] Suttle, CA.: Cyanophages and their role in the ecology of cyanobacteria. Ecol. Cyanobacteria. 563-589 (2000). 10.1007/0-306-46855-7-20

[18] Frederickson CM, Short SM, Suttle CA (2003). The physical environment affects cyanophage communities in British Columbia inlets. Microb Ecol.

[19] Singh P, Singh SS, Srivastava A, Singh A, Mishra AK (2012) Structural functional and molecular basis of cyanophage-cyanobacterial interactions and its significance. Afr J Biotechnol 11(11). 10.5897/ajb10.790

[20] Traving SJ, Clokie MRJ, Middelboe M (2013). Increased acidification has a profound effect on the interactions between the cyanobacterium *Synechococcus* sp. WH7803 and itsviruses. FEMS Microb Ecol.

[21] Finke JF, Suttle CA (2019). The environment and cyanophage diversity: insights from environmental sequencing of DNA polymerase. Front Microbiol.

[22] Wilhelm SW, Weinbauer MG, Suttle CA, Jeffrey WH (1998). The role of sunlight in the removal and repair of viruses in the sea. Limnol Oceanogr.

[23] Cheng K, Zhao Y-J, Du X, Zhang Y, Lan S, Shi Z (2007). Solar radiation-driven decay of cyanophage infectivity and photoreactivation of the cyanophage by host cyanobacteria. Aquat Microb Ecol.

[24] Hurwitz BL, Brum JR, Sullivan MB (2015). Depth-stratified functional and taxonomic niche specialization in the ‘core’ and ‘flexible’ Pacific Ocean Virome. ISME J.

[25] Adriaenssens EM, Cowan DA (2014). Using signature genes as tools to assess environmental viral ecology and diversity. Appl Environ Microbiol.

[26] Dreher TW, Brown N, Bozarth CS, Schwartz AD, Riscoe E, Thrash C, Bennett SE, Tzeng SC, Maier CS (2011). A freshwater cyanophage whose genome indicates close relationships to photosynthetic marine cyanomyophages. Environ Microbiol.

[27] Ruiz-Perez CA, Tsementzi D, Hatt JK, Sullivan MB, Konstantinidis KT (2019). Prevalence of viral photosynthesis genes along a freshwater to saltwater transect in Southeast USA. Environ Microbiol Rep.

[28] Wang G, Murase J, Asakawa S, Kimura M (2009). Novel cyanophage photosynthetic gene *psb*A in the floodwater of a Japanese rice field. FEMS Microbial Ecol.

[29] Lindell D, Jaffe JD, Johnson ZI, Church GM, Chisholm SW (2005). Photosynthesis genes in marine viruses yield proteins during host infection. Nature.

[30] Clokie MRJ, Mann NH (2006). Marine cyanophages and light. Environ Microbiol.

[31] Millard AD, Zwirglmaier K, Downey MJ, Mann NH, Scanlan DJ (2009). Comparative genomics of marine cyanomyoviruses reveals the widespread occurrence of Synechococcus host genes localized to a hyperplastic region: implications for mechanisms of cyanophage evolution. Environ Microbiol.

[32] Chénard C, Suttle CA (2008). Phylogenetic diversity of sequences of cyanophage photosynthetic gene *psb*A in marine and freshwaters. Appl Environ Microbiol.

[33] Gao E-B, Gui J-F, Zhang Q-Y (2012). A novel cyanophage with a cyanobacterial nonbleaching protein A gene in the genome. J Virol.

[34] Nakamura G, Kimura S, Sako Y (2014). Genetic diversity of *Microcystis* cyanophages in two different freshwater environments. Arch Microbiol.

[35] Driscoll CB, Otten TG, Dreher TW (2016) Genome sequencing of two novel Ma-LMM01-like strains reveals patterns of conservation and divergence in a globally distributed *Microcystis* phage type. Comparative Genomics of Freshwater Bloom-Forming Cyanobacteria and Associated Organisms. Oregon State University Graduate School 102–138. https://ir.library.oregonstate.edu/concern/graduate_thesis_or_dissertations/rb68xf16d

[36] Takashima Y, Yoshida T, Yoshida M, Shirai Y, Tomaru Y, Takao Y, Hiroishi S, Nagasaki K (2007). Development and application of quantitative detection of cyanophages phylogenetically related to cyanophages Ma-LMM01 infecting *Microcystis aeruginosa* in fresh water. Microbes Environ.

[37] Kimura-Sakai S, Sako Y, Yoshida T (2015). Development of a real-time PCR assay for the the quantification of Ma-LMM01-type *Microcystis* cyanophages in a natural pond. Lett Appl Microbiol.

[38] Kimura S, Yoshida T, Hosoda N, Honda T, Kuno S, Kamiji R, Hashimoto R, Sako Y (2012). Diurnal infection patterns and impact of *Microcystis* cyanophages in a Japanese pond. Appl Environ Microbiol.

[39] Yoshida-Takashima Y, Yoshida M, Ogata H, Nagasaki K, Hiroishi S, Yoshida T (2012). Cyanophage infection in the bloom-forming cyanobacteria *Microcystis aeruginosa* in surface freshwater. Microbes Environ.

[40] Wang X, Jing R, Liu J, Yu Z, Jin J, Liu X, Wang X, Wang G (2016). Narrow distribution of cyanophage *psb*A genes observed in two paddy waters of Northeast China by an incubation experiment. Virol Sin.

[41] Zhong X, Jacquet S (2013). Prevalence of viral photosynthetic and capsid protein genes from cyanophages in two large and deep perialpine lakes. Appl Environ Microbiol.

[42] Mankiewicz-Boczek J, Jaskulska A, Pawełczyk J, Gagała I, Serwecinska L, Dziadek J (2016). Cyanophage infection of *Microcystis* bloom in lowland dam reservoir of Sulejów Poland. Microb Ecol.

[43] Rozon RM, Short SM (2013). Complex seasonality observed amongst diverse phyto- plankton viruses in the Bay of Quinte an embayment of Lake Ontario. Freshw Rev.

[44] Kokociński M, Gągała I, Jasser I, Karosiene J, Kasperoviciene J, Kobos J, Koreiviene J, Soininen J, Szczurowska A, Woszczyk M, Mankiewicz-Boczek J (2017). Distribution of invasive *Cylindrospermopsis raciborskii* in the East-Central Europe is driven by climatic and local environmental variables. FEMS Microbiol Ecol.

[45] Hach (1997) Water Analysis Handbook 3rd ed. HACH Company Loveland Colorado U.S.A. 1309

[46] Golterman HL, Glymo RS, Ohnstad MAM (1978). Methods for physical and chemical analysis of fresh waters. Blackwell Scientific Oxford Hydrobiologie und Hydrographie.

[47] Komárek J, Anagnostidis K (2005) Cyanoprokaryota, part 2. *Oscillatoriales*. In Süsswasser Flora von Mitteleuropa Band 19/2; Büdel B, Gärtner G, Krienitz L, Schagerl M, Eds.; Gustav Fischer: Jena, Germany 1–759

[48] Komárek J (2013) Cyanoprokaryota. 3. Heterocytous genera. In Süswasserflora von Mitteleuropa/Freshwater Flora of Central Europe; Büdel B, Gärtner G, Krienitz L, Schagerl M, Eds.; Springer: Berlin, Germany 1–1130

[49] Komárek J, Anagnostidis K (1999): Cyanoprokaryota. *Chroococcales*. Süßwasserflora von Mitteleuropa 19 (1). – Jena–Stuttgart–Lübek–Ulm

[50] Wetzel RG, Likens GE (2000). Limnological analyses.

[51] Giovannoni SJ, DeLong EF, Schmidt TM, Pace NR (1990). Tangential flow filtration and preliminary phylogenetic analysis of marine picoplankton Appl. Environ Microbiol.

[52] Mankiewicz-Boczek J, Izydorczyk K, Jurczak T (2006) Risk assessment of toxic cyanobacteria in Polish water bodies. In A. G. Kungolos C. A. Brebbia C. P. Samaras V. Popov (Eds.) Environmental toxicology. WIT Transactions on Biomedicine and Health WITpress Southampton Boston 10 49

[53] Hammer Ø, Harper DAT, Ryan PD (2001) PAST: paleontological statistics software package for education and data analysis. Palaeontologia Electronica 4(1) art. 4: 9

[54] Janžekoviĉ F, Novak T (2012) PCA – a powerful method for analyze ecological niches. In: Sanguansat, P. (Ed.), Principal component analysis – multidisciplinary applications. InTech, Croatia. 10.5772/38538

[55] Kottek MJ, Grieser C, Beck B, Rudolf and F Rubel,  (2006). World Map of the Köppen-Geiger climate classification updated. Meteorol Z.

[56] Paerl HW (2008). Nutrient and other environmental controls of harmful cyanobacterial blooms along the freshwater-marine continuum. Adv Exp Med Biol.

[57] Salmaso N, Bernard C, Humbert JF, Akçaalan R, Albay M, Ballot A, Catherine A, Fastner J, Häggqvist K, Horecka M, Izydorczyk K, Köker L, Komárek J, Maloufi S, Mankiewicz-Boczek J, Metcalf JS, Quesada A, Quiblier C, Claude Yéprémian C (2017) Basic guide to detection and monitoring of potentially toxic cyanobacteria. In: J. Meriluoto L. Spoof and G.A. Codd [eds.] Handbook of Cyanobacterial Monitoring and Cyanotoxin Analysis. John Wiley & Sons Ltd The Atrium Chichester UK 46–69

[58] Schrader C, Schielke A, Ellerbroek L, Johne R (2012). PCR inhibitors – occurrence properties and removal. J Appl Microbiol.

[59] Ge X, Wu Y, Wang M, Wang J, Wu L, Yang X, Zhang Y, Shi Z (2013). Viral metagenomics analysis of planktonic viruses in East Lake Wuhan China. Virologica Sinica.

[60] Kelly L, Ding H, Huang KH, Osburne MS, Chisholm SW (2013). Genetic diversity in cultured and wild marine *cyanomyoviruses* reveals phosphorus stress as a strong selective agent. ISME J.

[61] Jover LF, Effler TC, Buchan A, Wilhelm SW, Weitz JS (2014). The elemental composition of virus particles: implications for marine biogeochemical cycles. Nat Rev Microbiol.

[62] Kokociński M, Stefaniak K, Mankiewicz-Boczek J, Izydorczyk K, Soininen J (2010). The ecology of the invasive cyanobacterium (*Nostocales Cyanophyta*) in two hypereutrophic lakes dominated by *Planktothrix* agardhii (*Oscillatoriales* Cyanophyta). Eur J Phycol.

[63] Toporowska M, Ferencz B, Dawidek J (2018) Impact of lake-catchment processes on phytoplankton community structure in temperate shallow lakes. Ecohydrology e2017 10.1002/eco.2017

[64] Karosienė, J, Savadova-Ratkus, K, Toruńska-Sitarz, A, Koreivienė, J, Kasperovičienė, J, Vitonytė, I, Błaszczyk, A, Mazur-Marzec, H.: First report of saxitoxins and anatoxin-a production by cyanobacteria from Lithuanian lakes. Eur. J. Phycol. 1–12 (2020). 10.1080/09670262.2020.1734667

[65] Mischke U, Nixdorf B (2003). Equilibrium phase conditions in shallow German lakes: how Cyanoprokaryota species establish a steady state phase in late summer. Hydrobiologia.

[66] Dolman AM, Rücker J, Pick FR, Fastner J, Rohrlack T, Mischke U, Wiedner C (2012). Cyanobacteria and cyanotoxins: the influence of nitrogen versus phosphorus. PLoS ONE.

