# Supplementary material for: Cyanophage Distribution Across European Lakes of the Temperate-Humid Continental Climate Zone Assessed Using PCR-Based Genetic Markers
Source: Microb Ecol. 2021 Jun 5;83(2):284–95. doi: 10.1007/s00248-021-01783-y (PMC8891191; doi:10.1007/s00248-021-01783-y)
Supplement: Supplementary file 1 — (DOCX 180 kb) [file 248_2021_1783_MOESM1_ESM.docx]

**Cyanophage distribution across European lakes of the temperate-humid continental climate zone assessed using PCR-based genetic markers**

Aleksandra Jaskulska^1^, Sigitas Šulčius^2^, Mikołaj Kokociński^3^, Judita Koreivienė^2^, Arnoldo Font Nájera^1, 4^, Joanna Mankiewicz-Boczek^4^

1 UNESCO Chair on Ecohydrology and Applied Ecology, Faculty of Biology and Environmental Protection, University of Łódź, 12/16 Banacha, 90-237 Łódź, Poland

2 Laboratory of Algology and Microbial Ecology, Nature Research Centre, 2 Akademijos, LT-08412 Vilnius, Lithuania.

3 Department of Hydrobiology, Adam Mickiewicz University, 6 Uniwersytetu Poznańskiego, 61-614 Poznań, Poland

4 European Regional Centre for Ecohydrology of the Polish Academy of Sciences, 3 Tylna, 90-364 Łódź, Poland

Correspondence

Joanna Mankiewicz-Boczek, European Regional Centre for Ecohydrology of the Polish Academy of Sciences, 3 Tylna, 90-364 Łódź, Poland. Email j.mankiewicz@erce.unesco.lodz.pl


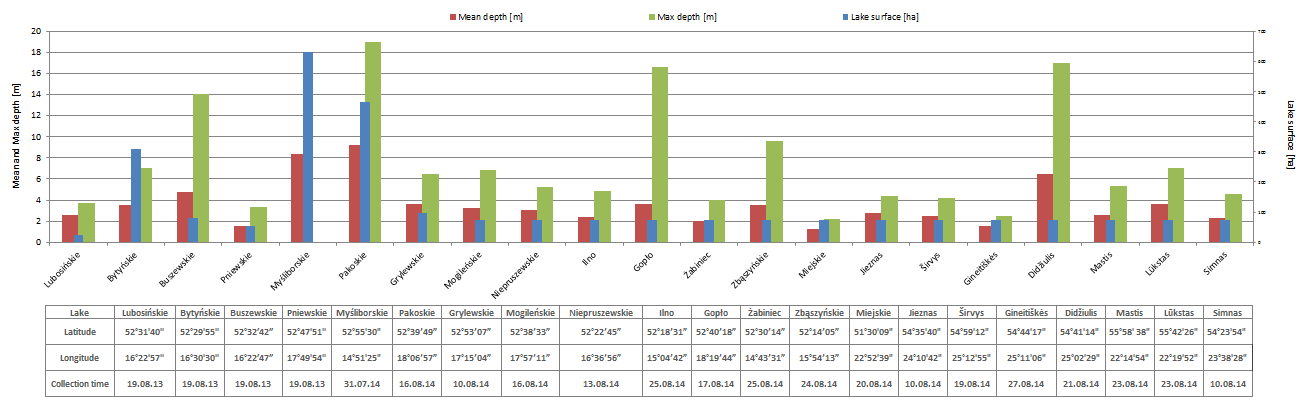


**Fig. S1** Basic information about investigated site eg. mean and max lakes depth, lakes surface, lakes latitude, lakes longitude, collection time

**Table S1** Physicochemical parameters and cyanobacterial composition in studied lakes.

| **Country** | **Lakes** | **Trophic state** | **pH** | **Cond [uS*cm^1-^]** | **Temp [°C]** | **TN [**mg*l^1-^**]** | **TP [**mg*l^1-^**]** | **TN:TP ratio** | **CYAN [mg l^-1^]** | **Dominant cyanobacteria and their biomass [mg L^-1^]*** |
| --- | --- | --- | --- | --- | --- | --- | --- | --- | --- | --- |
| **Poland** | LUB | eutrophic | 8.81 | 676.20 | 23.20 | 1.55 | 0.470 | 3 | 40.47 | *Planktothrix agardhii* 38.33 |
|  | BYT | eutrophic | 8.56 | 729.10 | 22.60 | 2.20 | 0.160 | 14 | 2.51 | *Microcystis aeruginosa* 1.35 |
|  | BUS | eutrophic | 8.78 | 588.10 | 23.30 | 2.29 | 0.250 | 9 | 13.30 | *Aphanizomenon gracile* 5.32, *Limnothrix redekei* 2.13,  *Limnothrix* sp. 2.20, *Planktothrix agardhii* 1.85 |
|  | PNI | eutrophic | 8.65 | 587.10 | 23.70 | 1.96 | 0.310 | 6 | 6.51 | *Aphanizomenon gracile* 2.28, *Raphidiopsis raciborskii* 1.43, *Jaaginema subtilissimum* 0.69, *Limnothrix redekei* 0.91 |
|  | MYS | eutrophic | 8.50 | 593.60 | 25.10 | 1.70 | 0.150 | 11 | 0.10 | *Aphanizomenon flos-aquae* 0.09 |
|  | PAL | eutrophic | 9.00 | 555.60 | 21.70 | 1.85 | 0.160 | 12 | 8.25 | *Planktothrix agardhii* 6.81 |
|  | GRY | eutrophic | 8.50 | 680.10 | 27.00 | 1.05 | 0.060 | 18 | 0.38 | *Raphidiopsis raciborskii* 0.06, *Jaaginema subtilissimum* 0.14, *Synechococcus salinarum* 0.15 |
|  | MOG | eutrophic | 7.40 | 596.80 | 21.60 | 2.45 | 0.020 | 123 | 13.70 | *Planktothrix agardhii* 10.75 |
|  | NIE | eutrophic | 8.60 | 681.70 | 23.60 | 7.50 | 0.130 | 58 | 2.73 | *Chrysosporum bergii* 0.37, *Jaaginema subtilissimum* 0.55, *Limnothrix vacuolifera* 0.83, *Planktolyngbya limnetica* 0.33 |
|  | ILN | eutrophic | 8.30 | 449.20 | 19.20 | 1.10 | 0.130 | 8 | 0.04 | *Jaaginema subtilissimum* 0.01*, Limnothrix redekei* 0.04 |
|  | GOP | eutrophic | 7.50 | 537.40 | 22.30 | 2.35 | 0.020 | 118 | 13.85 | *Planktothrix agardhii* 8.58, *Pseudanabaena limnetica* 1.46 |
|  | ZAB | eutrophic | 8.80 | 370.20 | 17.50 | 3.00 | 0.240 | 13 | 2.93 | *Dolichospermum lemmermannii* 0.42, *Dolichospermum planctonicum*0.44, *Aphanizomenon gracile* 0.81, *Microcystis* sp. 0.57 |
|  | ZBA | eutrophic | 9.00 | 618.50 | 17.20 | 2.80 | 0.060 | 47 | 37.48 | *Planktothrix agardhii* 36.35 |
|  | MIE | eutrophic | 8.70 | 326.00 | 21.40 | 1.50 | 0.050 | 30 | 10.31 | *Aphanocapsa* sp. 1.38, *Planktolyngbya limnetica* 5.60, *Radiocystis geminata* 1.10 |
| **Lithuania** | JIE | hypertrophic | 8.53 | 383.00 | 27.20 | 1.66 | 0.049 | 34 | 10.22 | *Pseudanabaena limnetica* 3.80, *Planktolyngbya limnetica* 3.41, *Microcystis viridis* 0.89, *Aphanizomenon gracile* 0.63 |
|  | SIR | hypertrophic | 7.81 | 447.00 | 20.50 | 1.28 | 0.055 | 23 | 3.83 | *Aphanizomenon gracile* 0.47, *Planktothrix agardhii* 1.23, *Pseudanabaena limnetica* 0.73 |
|  | GIN | eutrophic | 8.61 | 251.00 | 16.30 | 1.62 | 0.062 | 26 | 11,51 | *Woronichinia naegeliana* 2.47, *Planktolyngbya limnetica* 2.96, *Aphanizomenon gracile* 3.19 |
|  | DID | hypertrophic | 8.20 | 397.80 | 19.70 | 1.22 | 0.048 | 25 | 5.08 | *Aphanizomenon kelbanii* 0.73, *Planktolyngbya* sp. 2.27, *Pseudanabaena limnetica* 0.89 |
|  | MAS | hypertrophic | 8.40 | 334.00 | 17.00 | 1.15 | 0.160 | 7 | 6,13 | *Snowella* sp. 3.26, *Woronichinia compacta* 1.73 |
|  | LUK | meso-  eutrophic | 8.50 | 276.80 | 17.00 | 0.85 | 0.038 | 22 | 2.97 | *Planktolyngbya limnetica* 0.47*, Pseudanabaena limnetica* 0.89, *Woronichinia naegeliana* 0.56 |
|  | SIM | hypertrophic | 9.01 | 326.00 | 27.80 | 1.24 | 0.079 | 16 | 27.91 | *Aphanizomenon gracile* 5.99, *Cuspidothrix issachenkoi* 15.88 |

pH - water pH, Cond – water conductivity of water, Temp. - water temperature, TN - total nitrogen, TP - total phosphorus, CYAN - total biomass of cyanobacteria; *dominant cyanobacteria – indicated the strains which constituted 10% of total cyanobacteria biomass

| **TABLE S2** Sequences of primers used in presented study | | | | | | |
| --- | --- | --- | --- | --- | --- | --- |
| Biotic element | Targeting gene | Primer sequence (5'-3') | | Annealing temperature [°C] | Length (together with primer sequences) [bp] | References |
| Cyanobacteria | Cyanobacteria | 16SSF | CGGACGGGTGAGTAACGCGTG | 58 | 258 | 1 |
|  | 16S rRNA* | 16SSR | CCCATTGCGGAAAATTCCCC |  |  |  |
|  | *Microcystis* spp. | 209F | ATGTGCCGCGAGGTGAAACCTAAT | 51 | 250 | 2 |
|  | 16S rRNA | 409R | TTACAATCCAAAGACCTTCCTCCC |  |  |  |
| Cyanophages | *psb*A | Pro-psbA-1F | AACATCATYTCWGGTGCWGT | 50 | 740 | 3 |
|  |  | Pro-psbA-1R | TCGTGCATTACTTCCATACC |  |  |  |
|  | *nbl*A | nblARTF | GTGAGTGCCATTCCTGC | 55 | 200-211 | 4 |
|  |  | nblARTR | TCTTCTTGATGATAGCCGC |  |  |  |
|  | *g91_*S** | SheathRTF | ACATCAGCGTTCGTTTCGG | 56 | 132 | 5 |
|  |  | SheathRTR | CAATCTGGTTAGGTAGGTCG |  |  |  |
|  | *g91*_*L**** | g91 DRTPF1 | AGYGAGTTYCGCCTTAHTGT | 58 | 206 | 6 |
|  |  | g91 DRTPR2 | GRTGAYTGRCGTACYARRGC |  |  |  |
| ** universal 16S rRNA gene sequence for cyanobacteria* | | | | | | |
| *** g91 gene fragment with the lenght of 132 bp* | | |  |  |  |  |
| **** g91 gene fragment with the lenght of 206 bp* | | |  |  |  |  |

| **TABLE S3** Content of reagents used per PCR mixtures and description of PCR reaction conditions | | | | | | | | | | | | | | | | | | | | | | | | | | | | | | | | | | | | |
| --- | --- | --- | --- | --- | --- | --- | --- | --- | --- | --- | --- | --- | --- | --- | --- | --- | --- | --- | --- | --- | --- | --- | --- | --- | --- | --- | --- | --- | --- | --- | --- | --- | --- | --- | --- | --- |
| Target genes | | Cyanobacteria  16S rRNA** | |  | *Microcystis* spp.  16S rRNA | | |  | | *psb*A | | | | | |  | | *nbl*A | | | | |  | | *g91_*S*** | | | | |  | | *g91* gene_L****** | | | | |
| Content of reagents per one gene PCR mixture | PCR buffer | 1x | |  | 1x | | |  | | 1x | | | | | |  | | 1x | | | | |  | | 1x | | | | |  | | 1x | | | | |
|  | MgCl_2_ | 3 mM | |  | 3 mM | | |  | | 3 mM | | | | | |  | | 3 mM | | | | |  | | 3 mM | | | | |  | | 3 mM | | | | |
|  | Forward primers | 0.5 µM | |  | 0.5 µM | | |  | | 0.4 µM | | | | | |  | | 0.4 µM | | | | |  | | 0.25 µM | | | | |  | | 0.25 µM | | | | |
|  | Reverse primers | 0.5 µM | |  | 0.5 µM | | |  | | 0.4 µM | | | | | |  | | 0.4 µM | | | | |  | | 0.25 µM | | | | |  | | 0.25 µM | | | | |
|  | dNTPs | 0.2 mM | |  | 0.2 mM | | |  | | 0.2 mM | | | | | |  | | 0.2 mM | | | | |  | | 0.2 mM | | | | |  | | 0.2 mM | | | | |
|  | BSA | 0.1 mg ml1- | |  | 0.1 mg ml1- | | |  | | 0.1 mg ml1- | | | | | |  | | 0.1 mg ml1- | | | | |  | | 0.1 mg ml1- | | | | |  | | 0.1 mg ml1- | | | | |
|  | *Taq* polymerase | 1U | |  | 1U | | |  | | 0.85 U | | | | | |  | | 0.85 U | | | | |  | | 1 U | | | | |  | | 1 U | | | | |
| PCR steps | Temperature and time division | Temp [°C] | Time* |  | | Temp  [°C] | Time* |  | Temp  [°C] | | | | Time* | | |  | | Temp [°C] | | Time* | | |  | | Temp  [°C] | | Time* | | | |  | Tempe  [°C] | | | Time* | |
|  | Initial denaturation | 95 | 10 min |  | | 95 | 10 min |  | 95 | | | | 5 min | | |  | | 95 | | 5 min | | |  | | 95 | | 5 min | | | |  | 95 | | | 5 min | |
|  | Denaturation | 94 | 10 sec^1^ |  | | 94 | 10 sec^1^ |  | 95 | | | | 30 sec^2^ | | |  | | 95 | | 30 sec^2^ | | |  | | 94 | | 30 sec^3^ | | | |  | 94 | | | 30 sec^3^ | |
|  | Annealing of primers | 58 | 30 sec^1^ |  | | 51 | 30 sec^1^ |  | 55 | | | | 30 sec^2^ | | |  | | 55 | | 30 sec^2^ | | |  | | 56 | | 30 sec^3^ | | | |  | 56 | | | 30 sec^3^ | |
|  | Strand extension | 70 | 60 sec^1^ |  | | 70 | 60 sec^1^ |  | 72 | | | | 45 sec^2^ | | |  | | 72 | | 45 sec^2^ | | |  | | 72 | | 30 sec^3^ | | | |  | 72 | | | 30 sec^3^ | |
|  | Final extension | 72 | 10 min |  | | 72 | 10 min |  | 72 | | | | 5 min | | |  | | 72 | | 5 min | | |  | | 72 | | 10 min | | | |  | 72 | | | 10 min | |
| **min - minutes, sec - seconds* | | | | | | | | | | | | | | | | | | |  | |  |  | |  | |  | |  |  | | | |  |  | |  |
| *^1^ - stage repeated for 26 cycles* | | | | | | | | | | |  |  | |  |  | |  | |  | |  |  | |  | |  | |  |  | | | |  |  | |  |
| *^2^ - stage repeated for 34 cycles* | | | | | | | | | | |  |  | |  |  | |  | |  | |  |  | |  | |  | |  |  | | | |  |  | |  |
| *^3^ - stage repeated for 30 cycles* | | | | | | | | | | |  |  | |  |  | |  | |  | |  |  | |  | |  | |  |  | | | |  |  | |  |
| *** universal 16S rRNA gene sequence for cyanobacteria* | | | | | | | | | | |  |  | |  |  | |  | |  | |  |  | |  | |  | |  |  | | | |  |  | |  |
| **** g91 gene fragment with the lenght of 132 bp* | | | | | | | | | | |  |  | |  |  | |  | |  | |  |  | |  | |  | |  |  | | | |  |  | |  |
| ***** g91 gene fragment with the lenght of 206 bp* | | | | | | | | | | |  |  | |  |  | |  | |  | |  |  | |  | |  | |  |  | | | |  |  | |  |


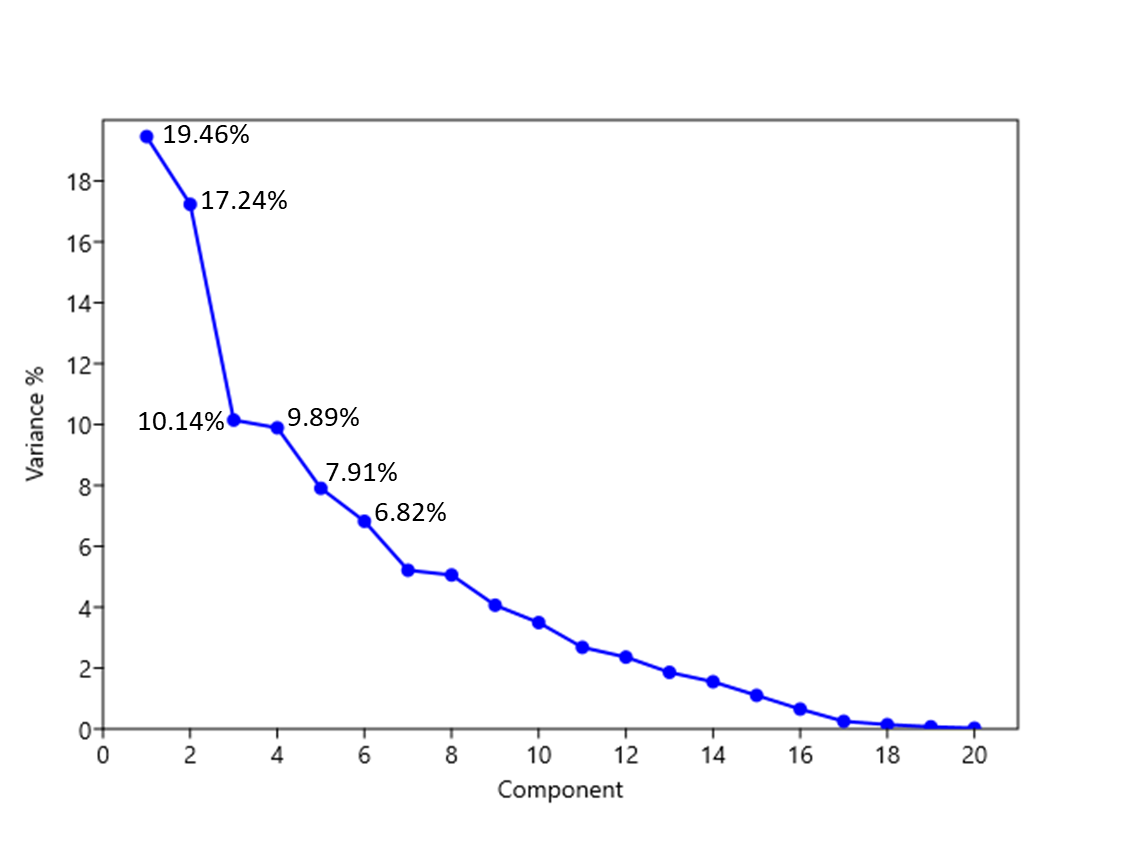


**Fig. S3** Summary of principal components and their total variance (%) to explain the spatial distance (PCA) between 21 Polish and Lithuanian lakes.

**Table S4** Description of PCA scores to plot the spatial distance between 21 Polish and Lithuanian lakes.

| **Country** | **Lake** |  | **PC 1** | **PC 2** | **Grouping** |
| --- | --- | --- | --- | --- | --- |
| Poland | LUB |  | 4.1856 | 0.95 | A |
|  | BYT |  | 1.7033 | 0.54 | A |
|  | BUS |  | 4.068 | 0.80 | A |
|  | PNI |  | 4.1476 | 0.82 | A |
|  | MYS |  | -0.88939 | -0.85 | C |
|  | PAL |  | 1.2041 | -0.08 | A |
|  | GRY |  | -0.1574 | -1.44 | C |
|  | MOG |  | -0.83694 | -2.21 | C |
|  | NIE |  | 2.0785 | -2.49 |  |
|  | ILN |  | -1.3378 | -1.38 | C |
|  | GOP |  | 0.48175 | -1.76 | C |
|  | ZAB |  | -0.34285 | -1.93 | C |
|  | ZBA |  | 0.8285 | -1.16 | C |
|  | MIE |  | -1.4343 | 1.37 |  |
| Lithuania | JIE |  | -2.9652 | 4.28 | B |
|  | SIR |  | -1.7371 | -1.69 | C |
|  | GIN |  | -1.4177 | 2.85 | B |
|  | DID |  | -1.7745 | -1.08 | C |
|  | MAS |  | -2.7993 | -0.60 |  |
|  | LUK |  | -3.1655 | -0.46 |  |
|  | SIM |  | 0.16068 | 5.50 | B |

**Table S5** Description of PCA loadings (Pearson correlation *r*) for the different environmental variables used to explain the spatial distancing between 21 Polish and Lithuanian lakes.

| **Parameter** | **Variable** |  | **PC 1** | **PC 2** |
| --- | --- | --- | --- | --- |
| Environmental factors | pH |  | 0.32826 | 0.46672 |
|  | Conductivity (Cond.) |  | 0.69807 | -0.38933 |
|  | Temperature (Temp.) |  | 0.32198 | 0.37879 |
|  | TN |  | 0.37191 | -0.33512 |
|  | TP |  | 0.71194 | 0.057194 |
|  | Cyanobacterial Biomass (CYAN) |  | 0.38034 | 0.35261 |
| Cyanophage genes | *psb*A |  | 0.51564 | 0.311 |
|  | *nbl*A |  | 0.56362 | 0.60583 |
|  | *g91*_S |  | 0.4231 | 0.79374 |
|  | *g91*_L |  | 0.22663 | 0.68897 |
| Cyanobacterial biomass | *Chrysosporum bergii* |  | 0.49199 | -0.062073 |
|  | *Aphanizomenon gracile* |  | 0.33695 | 0.62544 |
|  | *Cuspidothrix issatschenkoi* |  | 0.0082554 | 0.60458 |
|  | *Raphidiopsis raciborskii* |  | 0.62532 | -0.04905 |
|  | *Jaaginema subtilissimum* |  | 0.8514 | -0.18826 |
|  | *Limnothrix obliqueacuminata* |  | 0.36197 | -0.24887 |
|  | *Limnothrix redekei* |  | 0.38151 | 0.071305 |
|  | *Microcystis aeruginosa* |  | 0.071168 | 0.1485 |
|  | *Microcystis viridis* |  | -0.39936 | 0.50322 |
|  | *Microcystis wesenbergii* |  | -0.36868 | 0.71944 |
|  | *Planktolyngbya limnetica* |  | -0.32491 | 0.47156 |
|  | *Planktothrix agardhii* |  | 0.39348 | -0.095758 |
|  | *Pseudanabaena limnetica* |  | -0.41471 | 0.28758 |
|  | *Synechococcus salinarum* |  | 0.35222 | -0.23102 |
|  | *Woronichinia compacta* |  | -0.30411 | 0.17423 |
|  | *Woronichinia naegeliana* |  | -0.244 | 0.32206 |

**Table S6** Description of statistical analysis for the PC1

| **1) Test for equal means** | | | | | |
| --- | --- | --- | --- | --- | --- |
|  |  |  |  |  |  |
|  | Sum of sqrs | df | Mean square | F | p (same) |
| Between groups: | 54.8415 | 2 | 27.4208 | 18.77 | 1.09E-04 |
| Within groups: | 20.4513 | 14 | 1.4608 | Permutation p (n=99999) | |
| Total: | 75.2928 | 16 | 0.00059 |  |  |
|  |  |  |  |  |  |
|  |  |  |  |  |  |
| **2) Levene´s test for homogeneity of variance, from means** | | | | | |
| p (same): | 0.2119 | Samples are normaly distributed | | |  |
| 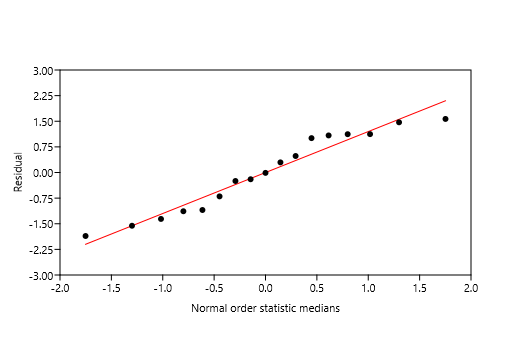   \| Residuals: \| \| --- \| |  |  |  |  |  |
|  |  |  |  |  |  |
|  |  |  |  |  |  |
|  |  |  |  |  |  |
|  |  |  |  |  |  |
|  |  |  |  |  |  |
|  |  |  |  |  |  |
|  |  |  |  |  |  |
|  |  |  |  |  |  |
| **3) Tukey’s test** | | | | | |
|  | **Group A** | **Group B** | **Group C** |  |  |
| **Group A** |  | 4.76E-04 | 2.20E-04 |  |  |
| **Group B** | 7.16 |  | 0.618 |  |  |
| **Group C** | 7.767 | 1.346 |  |  |  |
|  |  |  |  |  |  |
|  | Group A is significantly different from B and C | | | |  |

Significance *p* < 0.05 is highlighted in red.

**Table S7** Description of statistical analysis for the PC2

| **1) Test for equal means** | | | | | |
| --- | --- | --- | --- | --- | --- |
|  |  |  |  |  |  |
|  | Sum of sqrs | df | Mean square | F | p (same) |
| Between groups: | 74.984 | 2 | 37.492 | 91.76 | 8.99E-09 |
| Within groups: | 5.72052 | 14 | 0.408609 | Permutation p (n=99999) | |
| Total: | 80.7046 | 16 | 1.00E-05 |  |  |
|  |  |  |  |  |  |
|  |  |  |  |  |  |
| **2) Levene´s test for homogeneity of variance, from means** | | | | | |
| p (same): | 0.06219 | p > 0.05, then data is normaly distributed | | | |
| 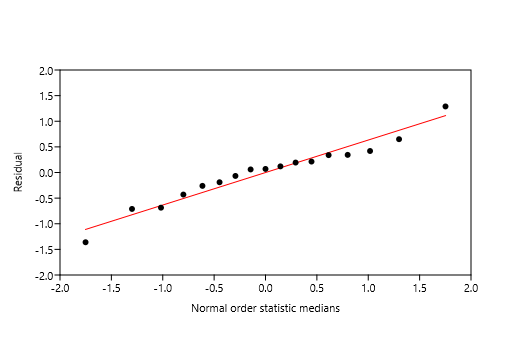   \| Residuals: \| \| --- \| |  |  |  |  |  |
|  |  |  |  |  |  |
|  |  |  |  |  |  |
|  |  |  |  |  |  |
|  |  |  |  |  |  |
|  |  |  |  |  |  |
|  |  |  |  |  |  |
|  |  |  |  |  |  |
|  |  |  |  |  |  |
| **3) Tukey’s test** | | | | | |
|  | **Group A** | **Group B** | **Group C** |  |  |
| **Group A** |  | 5.83E-06 | 1.06E-04 |  |  |
| **Group B** | 10.92 |  | 6.41E-09 |  |  |
| **Group C** | 8.353 | 18.95 |  |  |  |
|  |  |  |  |  |  |
|  | All groups are different between each other | | |  |  |

Significance *p* < 0.05 is highlighted in red.

**References**

1. Lin S, Shen J, Liu Y, Wu X, Liu Q, Li R (2011) Molecular evaluation on the distribution diversity and toxicity of *Microcystis* (Cyanobacteria) species from Lake Ulungur—a mesotrophic brackish desert lake in Xinjiang China Shen. Environ Monit Assess 175:139-150. https://doi.org/10.1007/s10661-010-1500-x

2. Neilan BA, Jacobs D, Del Dot T, Blackall LL, Hawkins PR, Cox PT, Goodman AE (1997) rRNA sequences and evolutionary relationships among toxic and nontoxic cyanobacteria of the genus *Microcystis*. Int J Syst Bacteriol 47(3):693-697 https://doi.org/0020-7713/97/ $04.00+0.

3. Sullivan MB, Lindell D, Lee JA, Thompson LR, Bielawski JP, Chisholm SW (2006) Prevalence and Evolution of Core Photosystem II Genes in Marine Cyanobacterial Viruses and Their Hosts PLoS Biology 4(8) e234. https://doi.org/10.1371/journal.pbio.0040234

4. Yoshida-Takashima Y, Yoshida M, Ogata H, Nagasaki K, Hiroishi S, Yoshida T (2012) Cyanophage Infection in the Bloom-Forming Cyanobacteria *Microcystis* *aeruginosa* in Surface Freshwater. Microbes Environ. 27(4):350–355

5. Takashima Y, Yoshida T, Yoshida M, Shirai Y, Tomaru Y, Takao Y, Hiroishi S, Nagasaki K (2007) Development and Application of Quantitative Detection of Cyanophages Phylogenetically Related to Cyanophages Ma-LMM01 Infecting *Microcystis* *aeruginosa* in Fresh Water. Microbes Environ 22(3):207–213.

6. Kimura-Sakai S, Sako Y, Yoshida T (2015) Development of a real-time PCR assay for the the quantification of Ma-LMM01-type *Microcystis* cyanophages in a natural pond. Letters in Apllied Microbiology 60:400-408. <https://doi.org/10.1111/lam.12387>
